# Medical and Physical Intelligence

**Published:** 1824-10

**Authors:** 


### MEDICAL AND PHYSICAL INTELLIGENCE.

### PATHOLOGY.

1. *Influence of the Stomach on the Production of Apoplexy.*—A MEMOIR on this subject, by J. R. RICHOND, has lately received the prize of the Royal Society of Medicine of Bordeaux. The following are the conclusions at which the author arrives:—

1. The stomach exercises a great influence on the brain; the close connexion which exists between them being indispensable to the execution of their functions.

2. The stomach expresses its wants to the brain, by the sensation of hunger and thirst; the former announces the necessity of excitement, the latter is the result of it.

3. Besides the functions of nutrition, which it effectually fulfils, the stomach likewise tends to keep the brain in the condition favourable for its action: it is, as it were, the balance of life.

4. The necessity of excitement, experienced by the mucous membrane of the digestive organs, is one of the most imperious, and one, the satisfying of which is the most necessary to the regularity of all the movements.

5. According as this excitement is more or less active, so is the action of the brain more or less energetic. To be convinced of this, it is only necessary to observe the effects of spirituous potations.

6. Drunkenness is the result of cerebral excitement, caused by the sympathetic action of the stimulated gastric membrane.

7. The ideas relative to taste are generally subordinate to the state of the mucous membrane of the digestive canal; and very frequently the moral is modified according to the sensations which the brain then perceives.

8. The influence of the stomach on the brain may be appreciated in the state of sleep, as well as watching.

9. In the state of disease, the stomach communicates the suffering to the brain; and from this participation results the development of cerebral phenomena, more or less perceptible to the observer.

10. These phenomena are not always purely sympathetic: the brain and its membranes are really affected more frequently than is supposed.

11. When the gastric irritation is chronic, and of long standing, there are almost always alterations in the brain or its coverings, which, by their sudden aggravation, may give rise to mania, epilepsy, and sometimes apoplexy.

12. This sudden aggravation which produces apoplexy, is most frequently the result of an excitement of the stomach communicated to the brain: it is in this way that it very frequently takes place during a repast, after indigestion, excess in spirituous liquors, the action of an emetic, &c.

13. The extravasation of blood may sometimes be the result of this, but its existence is only eventual, and it does not constitute the essence of the malady.

14. This extravasation, when it happens, is at the spot where the irritation predominated; because the vessels of this part, always gorged with the blood which flowed to it, have become dilated and softened, and thus rendered more liable to rupture.

15. If there be a partial softening of the brain, the blood may escape at this point, and produce those caverns which have so improperly been regarded as the product of hemorrhagy alone.

16. Very often, in chronic gastritis, we meet with evident alterations of the heart, which are not to be regarded as independent, but which are the result of the sympathetic transmission of the irritation of the stomach.

17. The treatment of apoplexy ought to be entirely antiphlogistic, for the disease is one of irritation.

18. Emetics, purgatives, and blisters, are, for the most part, injurious. When they succeed, it is by producing a revulsion; but this is rarely obtained, and most frequently the irritation which ought to produce it, turns to the increase of the disease it was intended to combat.

19. To attempt to procure this revulsion, would be to subject the patient to great danger, without much chance of success. It is easy, in truth, to conceive that a considerable alteration of the brain, or its membranes, cannot be cured in a rapid manner; and that the displacement of the irritation which has caused it must be very difficult, particularly if the means employed act upon a surface connected so intimately with the brain as the mucous membrane of the stomach.

20. All the substances which are regarded as proper to restore strength, to give tone, to diminish stupor, debility, &c. must be proscribed with care, so long as any irritation of the brain and stomach exists.

21. To have recourse to excitants, such as electricity, the nux vomica, &c. against consecutive paralysis, is to have an idea of the disease entirely false. These means cannot repair the ravages which exist in the brain, whilst they are of a nature to increase and aggravate them.

22. During convalescence, the stomach merits very particular attention: the practitioner ought never to forget that too much stimulation may produce fatal mischief. The lightest diet, mild drinks, an exact observance of all the principles of hygiene, and the removal of the causes capable of exciting the stomach and brain,—such are the means most proper to promote a recovery.

23. The employment of the means proper to calm cerebral and gastric irritation, which generally exist in persons having a predisposition to apoplexy, is the only anti-apoplectic to be trusted.—(*Bulletin des Sciences Medicales*, Juillet.)

2. *Palsy of the Lower Extremities cured without the assistance of Art.*—In a recent Number of a Journal called "Hippocrates," published at Rotterdam, and professing to embrace every branch of medical science, the case of a young girl is mentioned, who, after a fever, accompanied with affections of the nerves, had the inferior parts of the body paralysed: the legs were very much contracted; and this state of matters appears to have lasted for some years. In her seventeenth year, after having menstruated for the first time, the use of her limbs was suddenly restored. The author (Dr. HELLER) expresses his astonishment that the knee-joints had not been attacked with anchyloses during so long a period.—(*Hippocrates, Magazijn Toegewijd aan den Geheelen omvang van de Geneeskunde*, 1823.)

3. *Symptoms of Phthisis brought on from the presence of a foreign Body.*—A woman, about fifty years of age, had all the symptoms of phthisis, for which she was treated without success. All at once she brought up a fragment of bone by coughing, which she recollected having swallowed eight months before, when eating soup. She voided a pint and a half of purulent matter in the course of twenty-four hours, and became perfectly restored to health. Dr. S. LUISCIUS, who relates the case, puts as a query—where was the bone lodged?—(*Ib.*)

MIDWIFERY.

4. *Cases of Sudden Parturition.*—Mrs. M., a poor woman, aged twenty-seven, being in the habit of assisting her husband in the dredging business, like many other females here (Milford Haven), had spent the whole of this day (Oct. 15, 1823,) labouring on the water, although in the ninth month of pregnancy. On arriving home in the evening, and stepping from the boat to the shore, without any premonitory symptom whatever, she was delivered of a fine boy, which actually fell on the beach. The mother picked it up, carried it to her cottage, a distance of a quarter of a mile; and neither the babe nor herself sustained any inconvenience.—N. B. The inhabitants of the village where she resides have generally quick labours, and are only one remove from a state of nature in their habits.

Mrs. D., a middle-aged woman, in the ninth month of her pregnancy, having dressed herself for breakfast on the morning of February 17th, 1824, was in the act of coming down stairs, when suddenly a pain took her, she fell on her knees, and was delivered of a female child, before any one could arrive to her assistance. In this case the child was still-born; nor could I by any means animate it, although I persevered more than an hour.—(*Private letter from* W. THOMAS, Esq.)

MISCELLANEOUS.

5. *New Regulations for granting Degrees in Medicine, in the University of Edinburgh.*—We have been favoured with the perusal of a printed paper, entitled "Regulations for granting Degrees in Medicine, proposed by the Medical Faculty to the Senatus Academicus, July, 1824, and to be decided on by the Senatus in October, 1824."

This paper has caused a great "sensation" in the northern metropolis: it has given birth to several smart pamphlets, and to a whole host of controversial letters in the columns of the Edinburgh newspapers. Our readers must naturally feel a degree of interest in the questions which it agitates; some as being already the faithful *alumni* of that University; others from looking forward to it, as the source of their future honours; a few out of regard to a school which has contributed so essentially to the advancement of medical science; and the remainder out of mere curiosity. For their sakes we shall attempt a hasty analysis of the projected regulations, accompanied by such reflections as have occurred to us during their perusal; and we shall be happy if any observations of ours may assist the Senatus Academicus in their deliberations on the subject, which are, we find, to take place during the present month. The changes proposed in the mode of obtaining degrees in Edinburgh are reducible to the eight following heads:—

1st. The writing of a Thesis is made optional.

2. The period of study is extended from three to four years.

3. The indulgence granted for study in a foreign University is restricted to one year.

4. Study in London is allowed to count for one year.

5. A six months' course of midwifery is added to the established routine of study, the "curriculum," as it is technically called.

6. A three months' course of chemical instruction is added to the six months previously required.

7. Attendance on an hospital for six months in two separate years, is now proposed; or, in lieu of this, attendance for six months as an hospital clerk, or pupil for nine months at a dispensary, or service in his Majesty's army or navy.

8. The examinations, instead of being altogether in Latin, as heretofore, are modelled after the fashion of the Company of Apothecaries in London;—that is to say, the student is examined, " first, as to his proficiency in medical Latin, and in the prescribing of medicines; and afterwards, in English, on the different branches of medical science."

Those of our readers who are acquainted with the old system of graduation in Edinburgh, will find here the traces of a very *radical reform*.

The circumstances which have rendered this necessary are quite unknown to us. That the University has not fallen off in point of numbers is apparent from this,—that 109 gentlemen received the degree of M. D. there on Monday, the 2d of August last; and, that it has fallen off in point of general character, the professors surely would not wish to insinuate. What, therefore, can have induced the Medical Faculty to make such changes as are here contemplated, we are at a loss to conjecture.

But, granting that the necessity of the case could be made apparent to us, it remains to inquire whether the projected alterations are calculated to add to the fame of the University,—to advance the dignity of the profession of physic,—or perceptibly to increase the quantum of learning with which each individual M. D. leaves his Alma Mater, to enter on the duties of his profession.

1st. Making the Thesis optional, is copied from the regulations of the University of Glasgow; but we doubt whether the Medical Faculty of Edinburgh have, in this instance, selected for imitation the best part of their sister University's proceedings. True it is, that a Thesis is often the production of a grinder;—true it is, that, though the *ipsissima verba* of the candidate himself, it often contains the merest commonplace. Still it is a stimulus to exertion: it gives the candidate a certain feeling of consequence; it makes him an author once at least in his life; and, above all, it constitutes a ground of distinction between the diploma of Edinburgh, and that of the Company of Apothecaries in London. A Thesis, however, is not necessarily such trash as this: many a one has proved the nucleus of works which have, in later life, stamped the author with the character of an acute reasoner or accurate observer. But, independent of all this, a Thesis is either a good thing or a bad thing. If it is really unnecessary, why not strike it out altogether? Why give the candidate an option about this, and deny him the same option in every other part of the whole system of graduation?

2d. The extension of the period of study from three to four years, without any corresponding extension of the necessary courses of instruction, appears to us to be only opening the door to idleness, on the part of the student, for the first year or two of his academical residence.

3d. To the third proposed change, we can see no objection.

4th. A caviller might argue against the fourth, that it makes the exercise of the highest privilege of the University dependent on the pleasure of the surgeons and apothecaries of London; for it expressly stipulates, that such lectures only will be held to constitute study in London, as qualify for examination by the College of Surgeons and Company of Apothecaries. Here we anticipate no small share of trouble to the Dean of Faculty in Edinburgh hereafter, from this regulation; and we respectfully suggest to the Senatus, before deciding on this point, to read with attention the circular of the College of Surgeons of London, published in this Journal, in the Number for June of the present year, and satisfy themselves that they understand it.

5th. A six months' course of midwifery is now considered necessary for the education of a physician. We have heard it rumoured that this change met with great opposition on the part of the Medical Faculty, and that it was, in fact, forced upon them by a vote of their patrons, the Town Council. If this be so, it would be ungenerous to reproach the Faculty with it; but we must say, the innovation does not a little surprise us. Attendance on midwifery lectures is not required, either by the College of Surgeons or Company of Apothecaries in London, though the members of both the one and the other practise that art and mystery. On the other hand, midwifery lectures are now to be insisted on by the Medical Faculty of Edinburgh, as essential to a physician's education, though they know well enough that no inconvenience has hitherto resulted from the omission of that portion of study; and that the merits of their graduates will never be judged of by their knowledge of, or deficiency in, the obstetric art, the world making a very proper distinction between the physician and the practising accoucheur.

The attempt to evade this argument, by tacking to the lectures on midwifery the diseases of women and children, is hardly worthy of notice. If the diseases of one sex, or of one period of life, require separate investigation, so, by parity of reasoning, should there be separate lectures for diseases of old age, diseases peculiar to sedentary trades, diseases of hot climates, diseases of the eyes and ears, &c. &c. &c. If any additional class or classes had been added to the "curriculum" of a physician's education, we should, with great deference, have suggested a course of dissections, a course of comparative anatomy, and a course of medical jurisprudence.

6th. To the sixth proposed change no reasonable objection can be made.

7th. The seventh appears to us a most unnecessary, as well as most cumbersome, addition to the University regulations. The object is to ensure attention to hospital practice, during at least two years. Surely this is sufficiently provided for in the previous regulations, which require that the student should have attended "clinical lectures for nine months, and the hospital or hospitals connected with them." But, even with all their nicety of wording, the Faculty have omitted the case of the student entering the Infirmary of Edinburgh for one year, from the first day of July. He will then have attended six months in two "separate" years, unless, indeed, something more is meant by the term *separate* than we are aware of. We cannot, for our own parts, properly understand what it is that service in his Majesty's navy or army, or attendance on a London dispensary, "relieving 2000 patients annually," is to be an equivalent for.

8th. The last, but not the least, in the projected alterations of the

Edinburgh Faculty of Physic, relates to the language in which the examinations for degrees are hereafter to be held. Whatever doubts we might have had on some particular points, we can have none here; and we do earnestly call upon the Senatus Academicus to pause before they sanction a measure calculated, in our opinion, to do irreparable mischief to the profession of the physician. If it be argued, that the sciences of chemistry and pharmacy have so grown up since the days of the Romans, that it is impossible to descant on such subjects in Latin; and that the compounds of iodine and chlorine bid defiance both to grammar and dictionary, let there be an examination on these branches of science in English; but let the student be examined in anatomy, in physiology, and in pathology, in that language in which Celsus wrote, and Harvey wrote, and Sydenham wrote; men, with whose works it is devoutly to be hoped the graduates of the University of Edinburgh are familiar. It is frivolous and vexatious to meet this objection by saying, that an examination is still to be held to ascertain the student's proficiency in "medical Latin." The answer to this is obvious. If he be properly skilled in "medical Latin," he must be quite competent to answer questions on the different branches of medical science in that language. As the regulations now stand, the examination in medical Latin will, in a few years, dwindle into a mere form; and ultimately the graduate of Edinburgh will be on a par, in point of classical acquirements, with the apothecary's apprentice in London. The natural consequence of this will be, that, from deficiency in Latin, he will be refused admission into the College of Physicians in London, to the discredit of the University of Edinburgh, and the degradation of the profession of the physician.

This is not an exaggerated picture; and we again intreat the Faculty of Edinburgh to reconsider this portion of their projected changes, and, if possible, to return to those good old days when their University stood at least as high in public esteem as it is likely to do under the "new regulations." If this be impossible,—if the die be cast, and a new system determined on, because the Professors are tired of the old one, then we would suggest to the Faculty to make it optional with the student, whether he be examined in English or Latin; and, at all events, to introduce a clause permitting foreigners to be examined in the Latin language.

These are all the observations we have to offer upon the proposed changes; and, in concluding, we cannot but remark, that the only important deficiency in the existing regulations remains without any provision; or, rather, that, from the addition of another year, it is rendered more likely than ever to admit of serious abuse. We allude to the fact that no efficient measures are adopted for securing the *actual residence* of the student: at present, it is notorious that any one, after having once matriculated, may spend the rest of the session in London, Paris, or where he pleases. This, indeed, requires change and reformation.

We have only one word more to say. The following interesting N.B. is appended to the new code:—"The Medical Faculty mean also to propose to the Senatus to petition government for a remission of the tax on medical degrees, imposed in 1809." It is impossible to read this without smiling,—it has so much the appearance of a bonus held out to the student, as a compensation for the additional expence attending the fourth year of study. It must be recollected, however, that government may possibly be quite satisfied with the existing order of things: nay, for aught we know, they may be very much inclined, when their attention is called to it, to *increase*, instead of taking off the said tax. If the University of Edinburgh, therefore, would take our humble advice, they would say nothing about it, drop the new regulations, and adhere stedfastly to the old system, of which we never heard any complaints (except that just alluded to), till the Faculty of Medicine themselves, by their own acts and deeds, seemed to hint that something was " rotten in the state of Denmark."

6. *Medical, Clerical, and General Life-Assurance Company.*—This Institution, we understand, has been opened, and the business commenced, with every prospect of success. For the particulars, we refer our readers to the advertisement upon the Wrapper.

### METEOROLOGICAL JOURNAL,

*From August 20, to September 19, 1824.*

By Messrs. WILLIAM HARRIS and Co. 50, Holborn, London.

| | MOON. | Rain gauge | THERM. | | | BAROM. | | DE LUC'S HYG. | | WIND. | | ATMOSPHERIC VARIATIONS. | | |
|---|---|---|---|---|---|---|---|---|---|---|---|---|---|---|
| | | | 9 A.M. | MAX. | MIN. | 9 A.M. | 10P.M. | 9 A.M. | 10P.M. | 9 A.M. | 10P.M. | 9 A.M. | 2 P.M. | 10P.M. |
| Aug. | | | | | | | | | | | | | | |
| 20 | | | 60 | 65 | 56 | 29.78 | 29.80 | 77 | 78 | W | W | Overc. | Cloud. | Cloud. |
| 21 | | | 60 | 65 | 52 | 29.74 | 29.80 | 80 | 78 | W | NW | Rain | Rain | Cloud. |
| 22 | | .56 | 55 | 65 | 50 | 30.00 | 30.07 | 78 | 80 | NE | N | | | Fine |
| 23 | | | 50 | 68 | 51 | 30.07 | 30.10 | 80 | 78 | N | NE | Fine | Fine | |
| 24 | ● | | 51 | 65 | 50 | 30.16 | 30.15 | 79 | 82 | NE | N | Overc. | | |
| 25 | | | 57 | 70 | 58 | 30.26 | 30.25 | 80 | 78 | E | ESE | Fine | | |
| 26 | | | 63 | 67 | 52 | 30.30 | 30.27 | 75 | 78 | NE | E | Fair | | |
| 27 | | | 65 | 67 | 56 | 30.25 | 30.12 | 75 | 75 | ENE | NE | Fine | | |
| 28 | | | 64 | 79 | 56 | 30.00 | 29.90 | 75 | 80 | ENE | E | | | |
| 29 | | | 54 | 78 | 55 | 29.87 | 29.87 | 80 | 76 | E | SW | | | |
| 30 | | | 60 | 76 | 58 | 29.87 | 29.83 | 84 | 80 | SW | NNE | Foggy | | |
| 31 | ☽ | | 59 | 72 | 59 | 29.87 | 29.92 | 85 | 80 | NE | NNE | Overc. | | |
| Sep. | | | | | | | | | | | | | | |
| 1 | | | 66 | 81 | 60 | 29.98 | 30.00 | 80 | 77 | E | SSW | Fine | | |
| 2 | | | 69 | 83 | 66 | 30.00 | 29.99 | 79 | 78 | ESE | NE | | | |
| 3 | | | 70 | 78 | 65 | 29.98 | 29.86 | 70 | 76 | ESE | WSW | Foggy | Overc. | Cloud. |
| 4 | | | 67 | 74 | 59 | 29.82 | 29.80 | 76 | 78 | W | WSW | Cloud. | | |
| 5 | | | 65 | 73 | 57 | 29.74 | 29.67 | 75 | 70 | WSW | SW | Fine | Fine | Fine |
| 6 | | | 62 | 70 | 57 | 29.50 | 29.45 | 77 | 77 | S | WSW | Cloud. | Sho'ry | Cloud. |
| 7 | | | 63 | 65 | 55 | 29.50 | 29.44 | 75 | 87 | SW | WSW | | | Fine |
| 8 | ○ | 145 | 53 | 62 | 50 | 29.40 | 29.41 | 85 | 82 | SW | W | Rain | Fine | Rain |
| 9 | | | 55 | 62 | 52 | 29.60 | 29.70 | 80 | 80 | W | NNE | Fine | Rain | |
| 10 | | .77 | 53 | 64 | 45 | 29.74 | 29.72 | 80 | 89 | NW | SE | Cloud. | | |
| 11 | | | 60 | 65 | 55 | 29.60 | 29.73 | 90 | 90 | SSW | SW | Fine | Sho'ry | Rain |
| 12 | | .70 | 57 | 65 | 52 | 29.57 | 29.80 | 88 | 83 | SW | SW | Rain | | Fine |
| 13 | | | 59 | 67 | 57 | 30.00 | 30.05 | 80 | 77 | WSW | SW | Fine | Fine | |
| 14 | | | 62 | 67 | 62 | 30.01 | 29.97 | 71 | 78 | S | SSW | Cloud. | | |
| 15 | | | 65 | 69 | 64 | 30.01 | 30.10 | 80 | 79 | SSW | W | Fine | | |
| 16 | ☾ | | 65 | 72 | 59 | 30.20 | 30.15 | 75 | 80 | W | SE | | | |
| 17 | | | 67 | 71 | 60 | 30.12 | 30.00 | 90 | 95 | E | SE | Foggy | | Mist |
| 18 | | | 65 | 72 | 58 | 30.03 | 29.95 | 95 | 90 | E | ESE | Misty | | Fine |
| 19 | | | 60 | 65 | 55 | 29.90 | 29.80 | 90 | 93 | W | W | Overc. | Fine | Overc. |

The quantity of rain fallen in the month of August, was 2 inches and 87.100ths.

